# The *Cytospora chrysosperma* Virulence Effector CcCAP1 Mainly Localizes to the Plant Nucleus To Suppress Plant Immune Responses

**DOI:** 10.1128/mSphere.00883-20

**Published:** 2021-02-24

**Authors:** Zhu Han, Dianguang Xiong, Zhiye Xu, Tingli Liu, Chengming Tian

**Affiliations:** a The Key Laboratory for Silviculture and Conservation of Ministry of Education, College of Forestry, Beijing Forestry University, Beijing, China; b Provincial Key Laboratory of Agrobiology, Jiangsu Academy of Agricultural Sciences, Nanjing, China; University of Georgia

**Keywords:** *Cytospora chrysosperma*, virulence effector, subcellular localization, plant immunity

## Abstract

Canker disease is caused by the fungus Cytospora chrysosperma and damages a wide range of woody plants, causing major losses to crops and native plants. Plant pathogens secrete virulence-related effectors into host cells during infection to regulate plant immunity and promote colonization. However, the functions of *C. chrysosperma* effectors remain largely unknown. In this study, we used Agrobacterium tumefaciens-mediated transient expression system in Nicotiana benthamiana and confocal microscopy to investigate the immunoregulation roles and subcellular localization of CcCAP1, a virulence-related effector identified in *C. chrysosperma*. CcCAP1 was significantly induced in the early stages of infection and contains cysteine-rich secretory proteins, antigen 5, and pathogenesis-related 1 proteins (CAP) superfamily domain with four cysteines. CcCAP1 suppressed the programmed cell death triggered by Bcl-2-associated X protein (BAX) and the elicitin infestin1 (INF1) in transient expression assays with Nicotiana benthamiana. The CAP superfamily domain was sufficient for its cell death-inhibiting activity and three of the four cysteines in the CAP superfamily domain were indispensable for its activity. Pathogen challenge assays in N. benthamiana demonstrated that transient expression of CcCAP1 promoted *Botrytis cinerea* infection and restricted reactive oxygen species accumulation, callose deposition, and defense-related gene expression. In addition, expression of green fluorescent protein-labeled CcCAP1 in N. benthamiana showed that it localized to both the plant nucleus and the cytoplasm, but the nuclear localization was essential for its full immune inhibiting activity. These results suggest that this virulence-related effector of *C. chrysosperma* modulates plant immunity and functions mainly via its nuclear localization and the CAP domain.

**IMPORTANCE** The data presented in this study provide a key resource for understanding the biology and molecular basis of necrotrophic pathogen responses to Nicotiana benthamiana resistance utilizing effector proteins, and CcCAP1 may be used in future studies to understand effector-triggered susceptibility processes in the *Cytospora chrysosperma*-poplar interaction system.

## INTRODUCTION

Cytospora chrysosperma, a pathogenic fungus that causes canker disease, attacks nearly 80 species of woody plants, including poplar (*Populus* sp.), causing serious forestry and ecological damage each year, especially in China (1–4). Until now, studies on this disease have been limited to epidemiology, histocytology, and taxonomy (5–7), with few molecular biology studies in progress (8). However, understanding the molecular mechanisms of pathogenesis is important for the development of strategies for durable and efficient control of plant diseases (9, 10). Thus, the molecular mechanisms used by *C. chrysosperma* for successful colonization need to be elucidated.

Plants possess a multifaceted innate immune system to guard themselves against phytopathogens (11). Pathogen-associated molecular patterns (PAMPs) induce PAMP-triggered immunity (PTI), the first layer of plant immunity known as a type of basal defense (12). PAMPs are evolutionarily conserved molecules, such as lipopolysaccharide, the translation elongation factor EF-Tu, and flagellin from bacteria (13, 14); β-glucan, chitin, and ergosterol from fungi (15–17); and transglutaminase GP42, cellulose-binding elicitor lectin, and the elicitin infestin1 (INF1) from oomycetes (18–20). PAMPs are often recognized by plants via pattern recognition receptors on the plasma membrane, including receptor-like kinases, receptor-like proteins, and receptor-like cytoplasmic kinases (21–23). This basal defense response can restrict the proliferation of most pathogens via callose deposition in the cell walls, reactive oxygen species (ROS) accumulation, and transcriptional upregulation of immune-related genes (23).

Furthermore, phytopathogens evade or overcome PTI for further colonization in the host by delivering effectors into the plant cytoplasm or apoplastic space (24–29). This results in effector-triggered susceptibility (11). However, when effectors are recognized by corresponding resistance (R) proteins in the host plants, effector-triggered immunity is induced, which is a qualitatively swifter and more vigorous immune response than PTI and induces localized programmed cell death (PCD) in the host, also called the hypersensitive response (HR) (30).

Dedicated research on effector functions is important to understand the pathogenesis of phytopathogens and to contribute to breeding efforts for improved protection from disease (31). The past few decades have seen great progress in our knowledge of the activity of effectors and targets in host plants, which revealed that effectors manipulate plant immunity in several ways (32–36). (i) For example, some effectors suppress the RNA silencing process in host plants. For instance, *Phytophthora* suppressors of RNA silencing 1 and 2 (PSR1 and PSR2, respectively) from *Phytophthora sojae* inhibited the production of small RNA to hinder plant resistance (37–39). (ii) Some effectors interfere with PTI. For instance, *Cladosporium fulvum* Ecp6, Ustilago maydis Pep1, and *Phytophthora infestans* Avr3a target chitin oligosaccharide, peroxidase, and plant ubiquitination protein degradation enzymes, respectively (28, 40–45). (iii) Some effectors inhibit the HR, which is primarily triggered by the recognition of effector proteins (termed Avr proteins) by R proteins. (iv) Effectors target diverse immune signaling pathways, such as mitogen-activated protein kinase (MAPK) (46) and Brassinosteroid insensitive 1-associated receptor kinase1 (BAK1) pathways (47). Fungal effectors can be used according to their different pathogenic strategies for the identification of key components of plant innate immunity and for disease resistance breeding (36). However, of the several hundred effectors potentially produced by each phytopathogen, only a few effectors have been functionally characterized.

The localization of effector proteins in host cells often provides important clues to their mode of action (36). Effectors can target specific plant compartments, such as the nucleus, cytoplasm, tonoplast, vacuole, endoplasmic reticulum, chloroplast, mitochondria, and even the plasmodesma (48–53), and this localization is important for achieving their functions (49, 50, 54–57). For instance, the effector PsAvh52 from *P. sojae* enhances susceptibility in soybean (Glycine max) by relocating the host cytoplasmic transacetylase GmTAP1 into nuclear speckles (58). Moreover, the RxLR effector Avh241 from *P. sojae* localizes to the plasma membrane to induce plant cell death (59), and a tonoplast-associated protein, HaRxL17 from *Hyaloperonospora arabidopsidis*, enhances plant susceptibility (50). Thus, investigation of the subcellular localization of an effector helps to uncover its mode of action in host cells (36, 60).

The CAP (cysteine-rich secretory proteins, antigen 5, and pathogenesis-related 1 protein) superfamily members are mostly secreted glycoproteins that are present in a wide range of organism kingdoms and implicated in a wide range of biological processes, such as reproduction, development, immune function, and pathogen virulence (61, 62). CAP superfamily proteins typically, but not always, contain a signal sequence directing proteins to the extracellular environment, where their disulfide-stabilized structure is presumably important for overall stability, or untypically to specific intracellular compartments. Moreover, all cysteine-rich secretory proteins (CRISPs) contain a predicted signal peptide consistent with their extracellular localization or their localization to specific intracellular compartments. Although they do not contain transmembrane domains, they are sometimes found associated with membranes potentially either through glycosylation or through interactions with integral membrane proteins. Uniquely, GLIPR2 proteins do not contain a predicted signal sequence. This is consistent with its intracellular localization to the Golgi membrane (61).

Functional analysis of the secreted CAP members Pathogen Related in Yeast PRY1 and PRY2 in Saccharomyces cerevisiae showed that they are involved in sterol binding and export of acetylated cholesterol, and more importantly, the CAP domain alone was sufficient for their functions (63, 64). And the defects of PRY mutants in S. cerevisiae could be restored by the human CAP protein CRISP2 and Candida albicans pathogenesis-related 1 (PR-1) proteins Rbe1 and Rbt4 which contribute to C. albicans pathogenicity in a redundant way and are able to specifically bind cholesterol *in vitro* (65), suggesting a conserved function of CAP domain containing proteins (63, 66). Recently, CAP proteins have also emerged as novel virulence factors in pathogenic fungi and nematodes. Apart from Candida albicans PR-1 proteins, studies on non-plant PR-1-like (PR-1L) proteins revealed four PR-1L proteins in Fusarium graminearum—FgPr-1l-1, FgPr-1l-2, FgPr-1l-3, and FgPr-1l-4—and provided the first example that pathogen-derived PR-1L protein affects host virulence during F. graminearum-wheat interaction (67). A well-characterized CAP protein Gr-VAP1 in *Globodera rostochiensis* acts as a virulence effector to target the papain-like cysteine protease Rcr3 and is recognized by the Cf-2 receptor, resulting in defense-related PCD in tomato (68). The finding that secreted CAP proteins are conserved fungal virulence factors suggests that they could serve as potential targets to reduce fungal infection (62).

Here, we used the Nicotiana benthamiana transient expression system to show that a virulence-associated effector, CCG_07874, from *C. chrysosperma* is induced upon infection and functions in the early stage of infection to suppress the PCD caused by Bcl-2-associated X protein (BAX) and INF1. CCG_07874 belongs to the CAP family and here was designated CcCAP1. The CAP superfamily domain of CcCAP1 was sufficient for its activity, and three of the four cysteine residues (C^154^, C^238^, and C^259^) were essential for its function. In addition, despite the localization of CcCAP1 in both the nucleus and the cytoplasm, only nuclear localization was sufficient for its manipulation activity. These results suggest a potential interaction mode for an effector in *C. chrysosperma*.

## RESULTS

### The CCG_07874 effector belongs to the CAP family and is highly induced at the early stage of infection on poplar.

**FIG 1:**
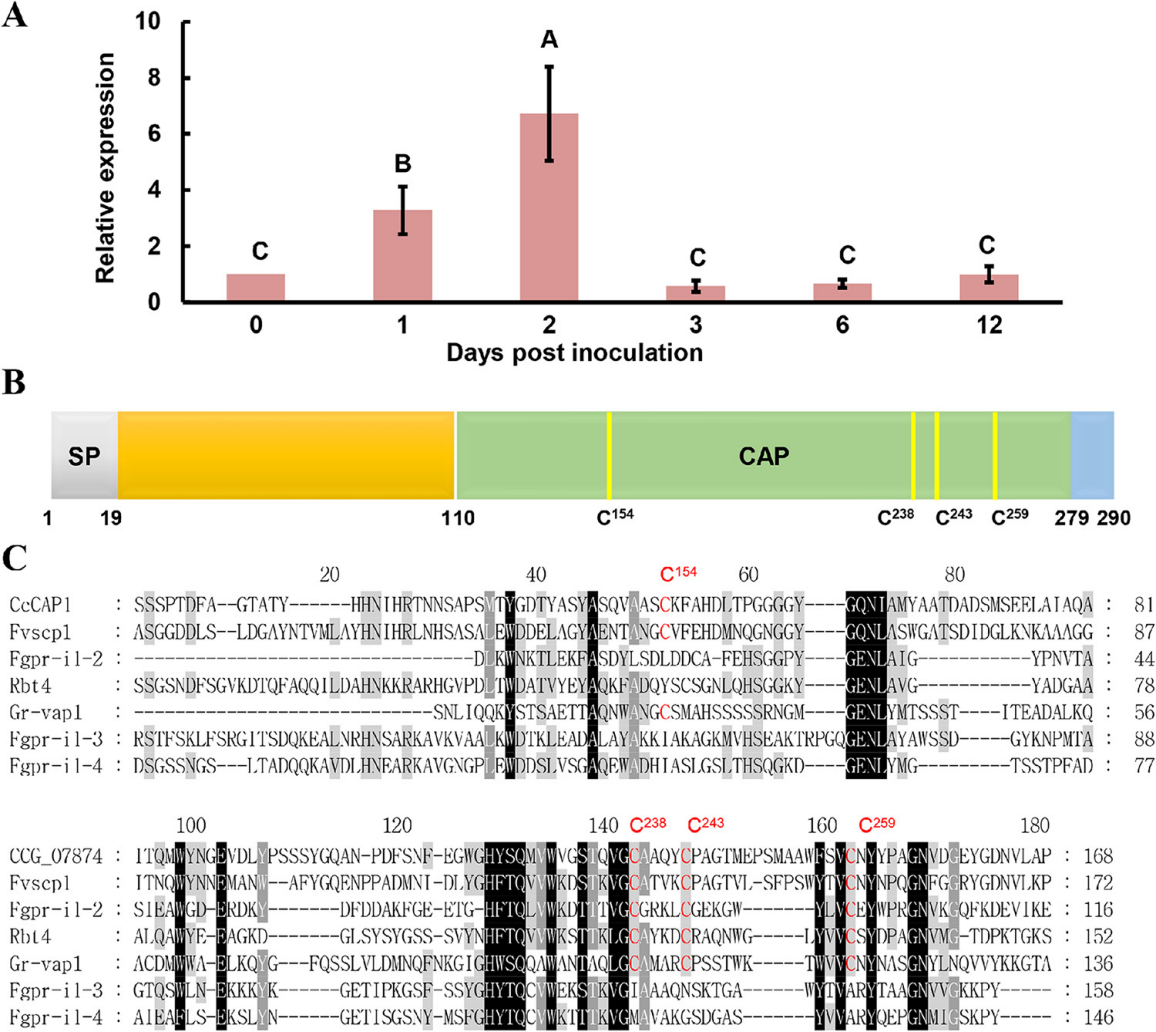
Significant upregulation of CcCAP1 at early infection stages. (A) Relative expression levels of the candidate effector CcCAP1 were detected at 0, 1, 2, 3, 6, and 12 dpi with the WT strain of *Cytospora chrysosperma* on poplar twigs, with *CcActin* as a reference gene. This experiment was performed three times. The statistical analyses were conducted by SPSS v16.0, and Duncan’s test at *P* = 0.05 was used to determine the differences. Bars indicate ± the standard errors (SE). Different letters indicate significant differences at *P* ≤ 0.01. (B) A schematic diagram of putative CcCAP1 architecture structure. SP, signal peptide, indicated in gray; CAP, the CAP domain, indicated in green; C, cysteine, indicated in yellow. (C) Sequence alignment of CcCAP1 CAP domain with six homologs. The cysteine residues conserved in these homologs are indicated in red.

The genomes of filamentous pathogens often encode hundreds of candidate effectors (69). In this study, nearly 300 candidate effector genes were identified in the *C. chrysosperma* genome (data not shown) based on general criteria, including their small sizes, the presence of a signal peptide in the N terminus, the lack of transmembrane domains, and being rich in cysteines, as described previously (70). Our previous study revealed an important pathogenesis-related MAPK, *C. chrysosperma* Pathogenicity MAP kinase 1 (CcPmk1), which regulates the expression of nine putative effectors (8). Among these effector candidates, CcCde3 (genome gene ID CCG_07874 [MN646886], identified by our lab) was selected for further study. CCG_07874 contains a cysteine-rich secretory protein, antigen 5, and pathogenesis-related 1 protein (CAP) superfamily domain (PF00188). CCG_07874 mRNA was significantly induced during the early stages of infection with an ∼3-fold increase at 1 day postinoculation (dpi) and a 7-fold increase at 2 dpi (Fig. 1A), which indicated that it might contribute to the colonization of *C. chrysosperma* during the early infection process. Sequence analysis showed that CCG_07874 contains 290 amino acids (aa) with four cysteine residues among the CAP domain and a predicted N-terminal signal peptide (SP; aa 1 to 18) (Fig. 1B).

To identify the putative functions of CCG_07874, we queried the CCG_07874 protein sequence against the PHI-base database. This identified six CAP homologs and showed that the CAP domain of the C terminus of CCG_07874 was conserved with proteins from various fungal pathogens and one nematode (Table 1), including FvSscp1 from F. verticillioides, Rbt4 from C. albicans, Gr-VAP1 from *G. rostochiensis*, and FgPr-1l-2, FgPr-1l-3, and FgPr-1l-4 from F. graminearum. Among these, Fvscp1, Rbt4, and FgPr-1l-4 were found to positively regulate the pathogen virulence, and Gr-vap1 acts as an avirulence effector (Table 1), suggesting that CCG_07874 may be a potent virulence effector acting during *C. chrysosperma*-plant interaction. Multiple sequence alignments of these homologs showed that the cysteine residues are also highly conserved (Fig. 1C).

**TABLE 1:** Homologs of CcCAP1 obtained by querying the CcCAP1 protein sequence against the PHIB-base database

No.	Gene	Species	Virulence	Length (aa)	E value	Reference
1	CcCAP1	Cytospora chrysosperma	Reduced virulence	290	0	This study
2	Fvscp1	Fusarium verticillioides	Reduced virulence	336	3.15 × 10^−44^	109
3	FgPr-1l-2	Fusarium graminearum	Unaffected pathogenicity	203	5.61 × 10^−14^	67
4	Rbt4	Candida albicans	Reduced virulence	358	3.11 × 10^−10^	65
5	Gr-vap1	Globodera rostochiensis	avirulence effector	219	9.07 × 10^−10^	68
6	FgPr-1l-3	Fusarium graminearum	Unaffected pathogenicity	268	1.88 × 10^−08^	67
7	FgPr-1l-4	Fusarium graminearum	Reduced virulence	246	0.04	67

In addition to CCG_07874, there are two other CAP members in *C. chrysosperma*, CCG_00371 and GME10144_g. Moreover, the three CAP members—CCG_07874, CCG_00371, and GME10144_g—in *C. chrysosperma* were designated CcCAP1, CcCAP2, and CcCAP3, respectively. CcCAP2 and CcCAP3 were also induced during *C. chrysosperma*-poplar interaction (data not shown). To study the phylogenetic distribution of the CAP superfamily domain in fungi, we selected the most common and well-studied CAP proteins in fungi, yeasts, and plants to construct the phylogenetic tree. As shown in Fig. S1 in the supplemental material, three CAP members of *C. chrysosperma* were distributed in three diverse clades. CcCAP1 fell into the clade 1 with proteins from *Fusarium* species, *B. cinerea*, and so on, while CcCAP2 clustered into a separate clade, designated clade 2, which is closer to the plant PR-1 proteins rather than CAP proteins from fungi. The third CAP member CcCAP3 was grouped into clade 3, which was phylogenetically closer to yeast CAP proteins. All sequences of CAP superfamily members from the selected species are listed in Table S1 in the supplemental material.

10.1128/mSphere.00883-20.1 FIG S1 Phylogenetic distribution of CcCAP1. The phylogeny of CAP members from selected species was assessed. The tree was constructed by the maximum-likelihood method. Bootstrap percentage support for each branch is indicated. All of the selected proteins were divided into three clades: clade 1, clade 2, and clade 3. Three CAP members of *C. chrysosperma*—CcCAP1, CcCAP2, and CcCAP3—are marked by a solid red circle. The clade CcCAP1 clustered into is circled in light gray, and plant PR-1 proteins are circled in dark gray. Download FIG S1, TIF file, 0.3 MB. Copyright © 2021 Han et al. 2021 Han et al. https://creativecommons.org/licenses/by/4.0/ This content is distributed under the terms of the Creative Commons Attribution 4.0 International license.

10.1128/mSphere.00883-20.5 TABLE S1 CAP superfamily members from selected species. Download Table S1, XLSX file, 0.02 MB. Copyright © 2021 Han et al. 2021 Han et al. https://creativecommons.org/licenses/by/4.0/ This content is distributed under the terms of the Creative Commons Attribution 4.0 International license.

In summary, CcCAP1 was selected as a candidate effector because it is induced in an early stage of infection, is highly conserved as a CAP member, has fewer than 300 aa, possesses a signal peptide, and is rich in cysteines.

### CcCAP1 mutants are significantly reduced in virulence and tolerance to H_2_O_2_.

To investigate the potential virulence role of CcCAP1, we generated CcCAP1 deletion mutants (ΔCcCAP1-2, ΔCcCAP1-4, ΔCcCAP1-6, ΔCcCAP1-8, and ΔCcCAP1-11) by replacing its full-length open reading frame sequence with a hygromycin cassette using the split-marker method (see Fig. S2A) and confirmed the deletions by PCR and Southern blot analysis (see Fig. S2B and C). CcCAP1 complementation strains (ΔCcCAP1/C-1 and ΔCcCAP1/C-2) were acquired by the same method, with the ΔCcCAP1-8 mutant as a recipient strain (see Fig. S2D).

10.1128/mSphere.00883-20.2 FIG S2 Deletion and complementation of CcCAP1. (A) Schematic diagram showing the deletion strategies of CcCAP1 by using the split-marker method. (B) The CcCAP1 deletion mutants were confirmed using PCR with the primer pairs Internal-CcCAP1for/Internal-CcCAP1rev and External-CcCAP1for/External-CcCAP1rev, respectively. Five CcCAP1 deletion mutants were successfully selected, ΔCcCAP1-2, -4, -6, -8, and -11. (C) Southern blot of the WT strain and two ΔCcCAP1 mutants (ΔCcCAP1-4 and ΔCcCAP1-8). DNA samples from the transformants were digested by SmaI. The enzyme-digested products were probed with a DNA sequence from the 5′ flanking sequence of CcCAP1. (D) ΔCcCAP1 mutants were complemented by inserting a fragment containing the full-length coding sequence of CcCAP1 flanking a 1.5-kb native promoter sequence upstream and a 0.2-kb terminator sequence downstream, respectively. The complemented mutants were analyzed by PCR with the primer pair Internal-CcCAP1for/Internal-CcCAP1rev. Download FIG S2, TIF file, 2.0 MB. Copyright © 2021 Han et al. 2021 Han et al. https://creativecommons.org/licenses/by/4.0/ This content is distributed under the terms of the Creative Commons Attribution 4.0 International license.

**FIG 2:**
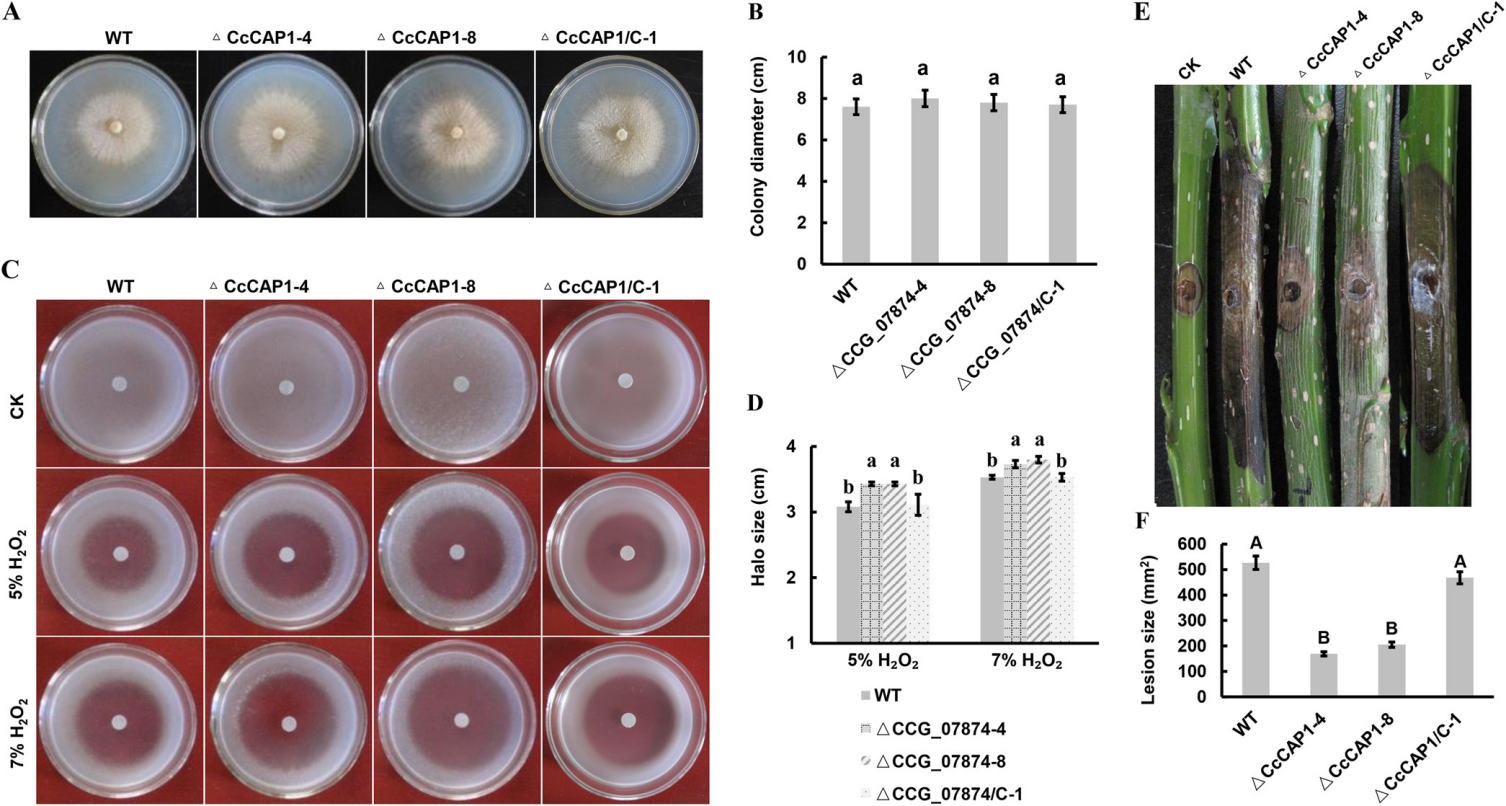
Indispensable role of CcCAP1 in ROS defense and pathogenicity. (A) Vegetative growth and morphological development of *C. chrysosperma* WT strain, ΔCcCAP1-4, ΔCcCAP1-8, and ΔCcCAP1/C-1 on PDA at 25°C for 3 days. (C) Colonial morphology of *C. chrysosperma* WT strain, ΔCcCAP1-4, ΔCcCAP1-8, and ΔCcCAP1/C-1 on PDA added with 5 and 7% H_2_O_2_ for 3 days, respectively. (E) Infection symptoms of *C. chrysosperma* WT strain, ΔCcCAP1-4, ΔCcCAP1-8, and ΔCcCAP1/C-1 on poplar twigs for 4 days. (B, D, and F) Quantification of colony diameter, halo size, and lesion area on the WT strain and on ΔCcCAP1-4-, ΔCcCAP1-8-, and ΔCcCAP1/C-1-treated media or twigs. This experiment was performed three times with similar results. Each assay was performed on at least three independent biological repeats. The statistical analyses were conducted by SPSS v16.0, and Duncan’s test at *P* ≤ 0.05 or *P* ≤ 0.01 was used for determining the differences between mutants and WT strain. Bars indicate ± the SE. The letters above the error bars indicate the different groups with statistical significance (*P ≤ *0.01 or *P ≤ *0.05).

To determine whether the CcCAP1 mutants affect the growth of *C. chrysosperma*, mycelial plugs of the wild-type (WT) strain, the deletion mutants ΔCcCAP1-4 and ΔCcCAP1-8, and the complementation strain ΔCcCAP1/C-1 were inoculated onto potato dextrose agar (PDA) plates at 25°C for 3 days in the dark. As shown in Fig. 2A and B, no obvious differences were observed in colony morphology and growth rate in the deletion mutant strains compared to the WT and complementation strains, indicating that the deletion in CcCAP1 did not affect the vegetative growth of *C. chrysosperma*.

The ROS burst, including an increase in hydrogen peroxide (H_2_O_2_), in host plants is an important strategy used by plants to suppress the infection of pathogens (71). Therefore, we investigated the role of CcCAP1 in the oxidative stress response of *C. chrysosperma* by adding conidial suspensions (1 × 10^6^ conidiospores/ml), which were harvested from mashed pycnidia of the WT, ΔCcCAP1 mutants, or complementation strains, in homogenized PDA medium and placing filter paper discs containing 5 μl of 5 or 7% H_2_O_2_ in the centers of the plates, as described previously (72). The deletions in CcCAP1 significantly reduced the tolerance of *C. chrysosperma* to H_2_O_2_ at 3 dpi compared to the WT and complementation strains (Fig. 2C and D).

Furthermore, we performed a pathogenicity test by inoculating poplar twigs from the susceptible species *Populus euramericana* with mycelial plugs of the WT strains, the deletion mutants, and the complementation strains. The WT and the complementation strains caused severe symptoms on the poplar twigs, but the poplar twigs inoculated with the CcCAP1 deletion mutants showed only slight symptoms (Fig. 2E and F).

These results suggested that CcCAP1 is an important factor in the virulence of *C. chrysosperma*.

### CcCAP1 suppresses BAX- and INF1-induced cell death in *N. benthamiana*.

**FIG 3:**
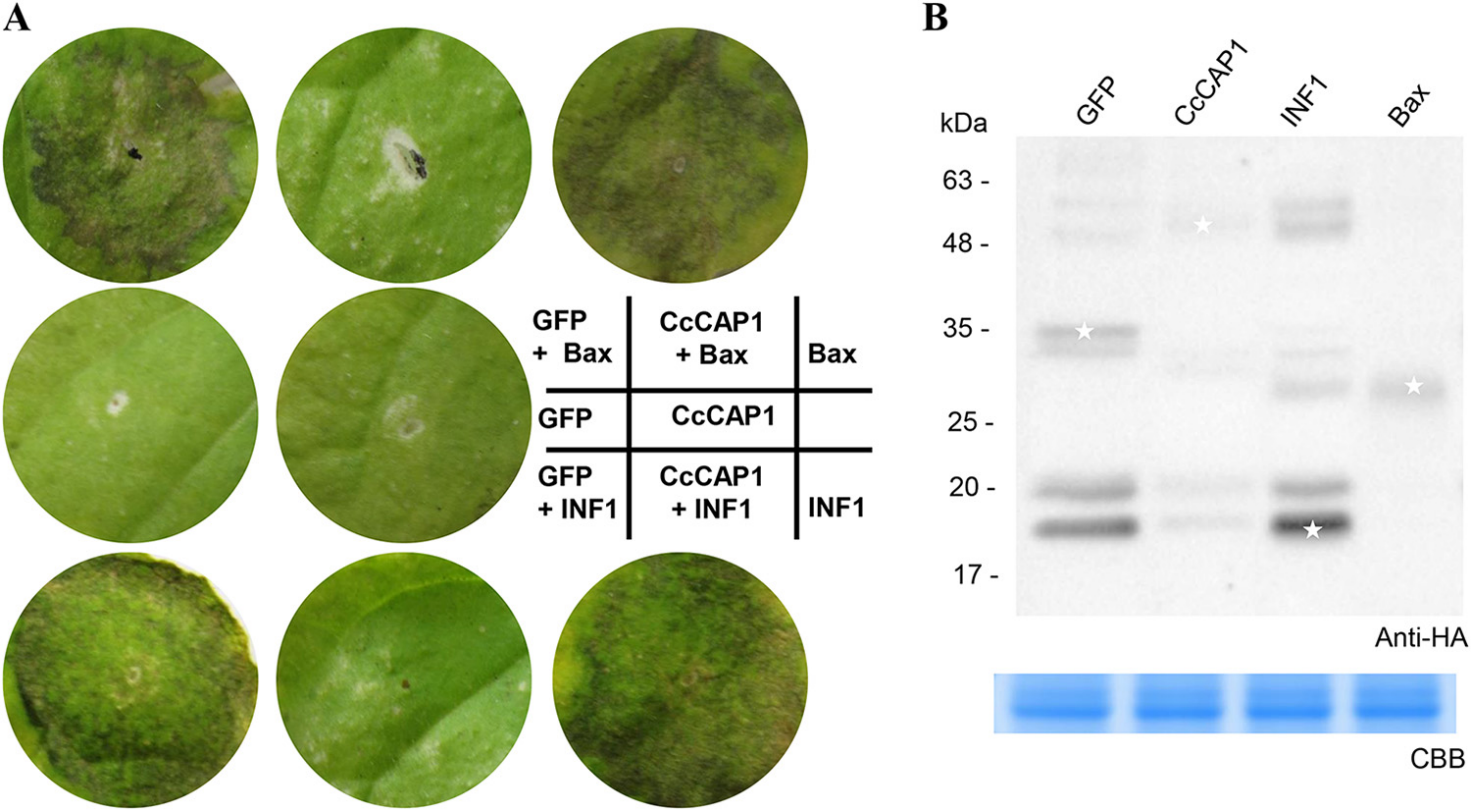
Inhibition of BAX- and INF1-induced cell death by transient expression of CcCAP1 in N. benthamiana leaves. (A) Representative symptoms on leaves of N. benthamiana were assessed at 5 dpa of CcCAP1-pGR106, with GFP-pGR106, BAX-pGR106, and INF1-pGR106 as a control. This experiment was performed at least three times with similar results. Each assay was performed on at least three plants. (B) Western blot analysis of proteins in *N. benthamiana* transiently expressing HA-tagged CcCAP1, GFP, Bax, and INF1. White asterisks indicate protein bands of interest.

Potato Virus X (PVX) agroinfection in N. benthamiana is a widely used and efficient transient expression assay for functional analysis of candidate effectors during the interaction between pathogens and plants. To assess the putative regulatory function of this pathogen effector in host plants, we tested whether CcCAP1 could induce necrosis or suppress PCD triggered by the mammalian proapoptotic factor BAX or the well-known oomycete PAMP INF1. BAX triggers PCD resembling the plant defense-related HR (73, 74), and INF1 strongly induces HR cell death. To test the effect of CcCAP1 on these processes, we used Agrobacterium tumefaciens-mediated transformation in N. benthamiana to transiently express BAX or INF1 (75–77) and coexpressed CcCAP1. As shown in Fig. 3A, the PCD induced by BAX or INF1 was almost totally blocked by coexpression with CcCAP1 compared to the green fluorescent protein (GFP) control, but CcCAP1 did not induce necrosis at 5 days after infiltration. The expression of hemagglutinin (HA)-tagged CcCAP1, GFP, Bax, and INF1 were confirmed by Western blotting (Fig. 3B). These results suggested that CcCAP1 is an important virulence-related effector of *C. chrysosperma* that is involved in manipulating plant immunity by suppressing cell death.

### The CAP domain is sufficient for the cell death-inhibiting activity of CcCAP1.

**FIG 4:**
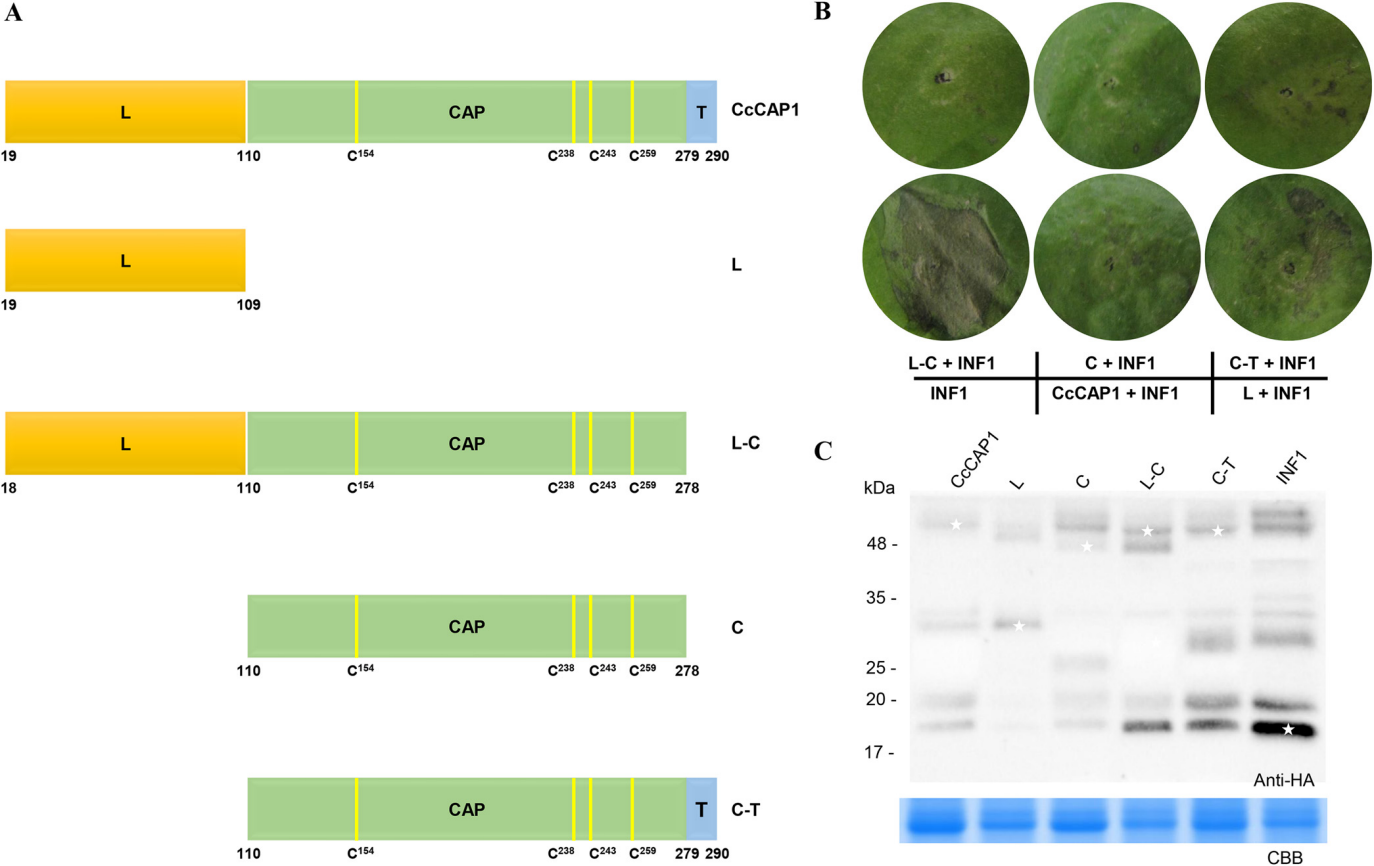
Significance of CAP domain in suppression of INF1-induced cell death. (A) Deletion mutants of CcCAP1. L, linker motif, indicated in orange; T, terminal motif, indicated in blue. (B) Deletion mutants were transient expressed by agroinfiltration in N. benthamiana to assay the suppression of INF1-induced cell death. Representative symptoms on leaves of N. benthamiana were photographed at 5 dpa. This experiment was performed at least three times with similar results. Each assay was performed on at least three plants. (C) Western blot analysis of proteins in *N. benthamiana* transiently expressing CcCAP1 and its variants fused with an HA tag. White asterisks indicate protein bands of interest.

To determine the functional regions in CcCAP1, we created four truncated domain mutants according to the domain structure by dividing the mature-type CcCAP1 into three components: the linker motif (aa 19 to 109, L), the CAP domain (aa 110 to 278, C), and the terminal motif (aa 279 to 290, T), and then we tested their ability to suppress cell death using the same transient expression system as described above (Fig. 4A). As shown in Fig. 4B, the truncated mutant of CcCAP1 lacking the CAP domain (L) could not inhibit the cell death induced by INF1, while mutants containing the full length of the CAP domain, including C-T, L-C, and C constructs (diagrammed in Fig. 4), could suppress the cell death induced by INF1. Transient expression of HA-tagged CcCAP1 and its variants were confirmed by Western blotting (Fig. 4C). These results suggested that the CAP domain of CcCAP1 from 110 to 278 aa is sufficient to inhibit the cell death induced by INF1.

### The cysteine residues in the CAP domain are required for suppression of cell death.

**FIG 5:**
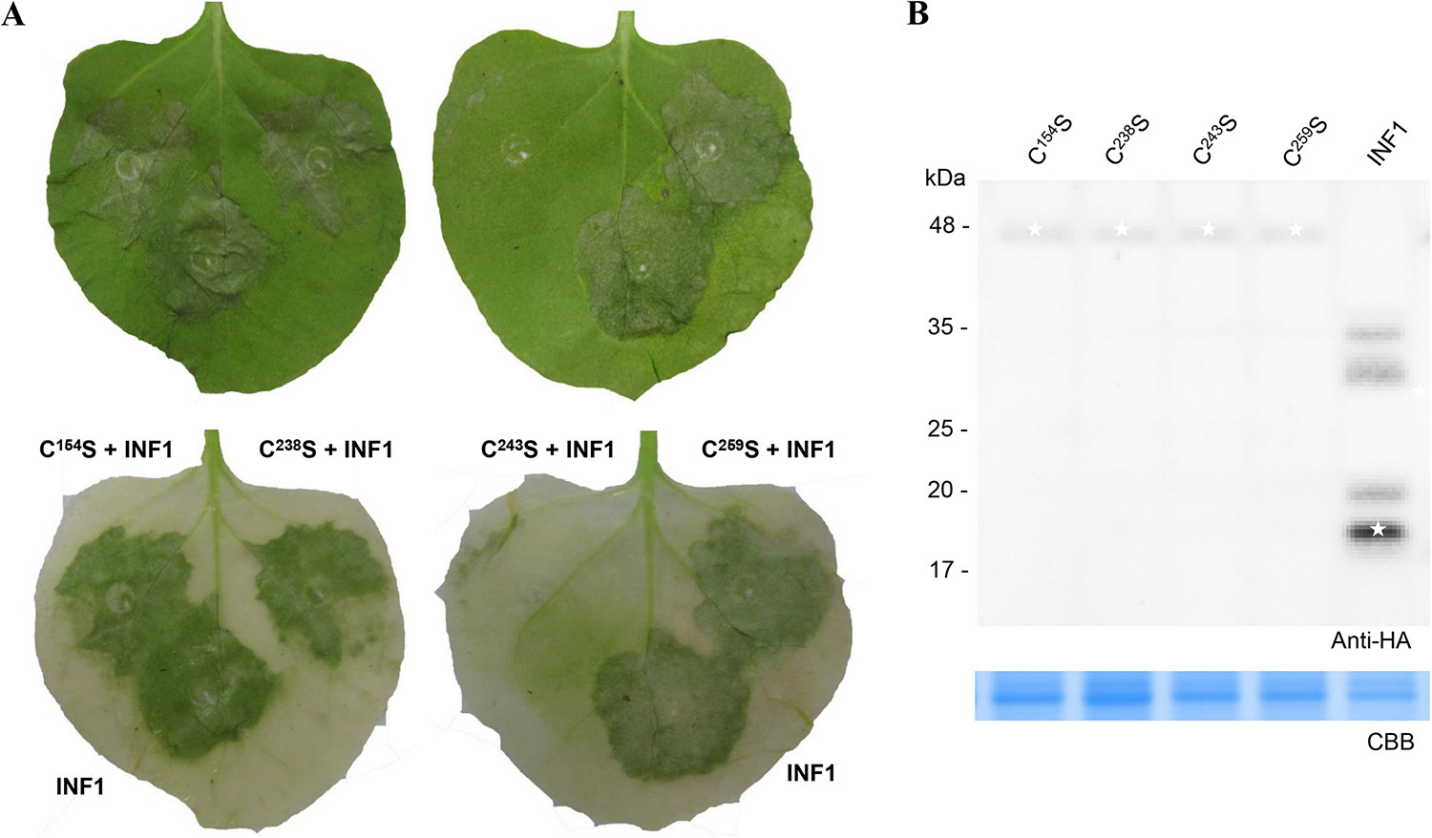
Determination role of the 154th, 238th, and 259th cysteine residues of CAP domain in suppression of INF1-induced cell death. (A) Representative symptoms on leaves of transient expressed CAP mutants CcCAP1-C^C154S^-pGR106 (C^154^S), CcCAP1-C^C238S^-pGR106 (C^238^S), CcCAP1-C^C243S^-pGR106 (C^243^S), and CcCAP1-C^C259S^-pGR106 (C^259^S) at 5 dpa. This experiment was performed at least three times with similar results. Each assay was performed on at least three plants. (B) Western blot analysis of proteins in *N. benthamiana* transiently expressing HA-tagged cysteine substitution mutants. White asterisks indicate protein bands of interest.

Cysteine residues are reported to be implicated in the formation of disulfide bridges that are thought to assist protein stability and affect protein function (78). As introduced above, there are four cysteine residues (C^154^, C^238^, C^243^, and C^259^) in the CAP domain of CcCAP1. However, no predicted disulfide bridges were found when searched in the Prosite database (https://prosite.expasy.org/) and Predictprotein database (https://predictprotein.org/). To test the putative roles of these four cysteine residues in the cell death suppression activity of the CAP domain, we individually replaced the four cysteine residues with serine using the single point mutation method. Then, all of the cysteine substitution mutants were examined using the *Agrobacterium* infiltration assay in N. benthamiana. Intriguingly, only the CAP^C243S^ mutant retained the full function of the CAP domain in inhibiting the cell death triggered by INF1, but the other three cysteine substitution mutants—CAP^C154S^, CAP^C238S^, and CAP^C259S^—completely lost activity (Fig. 5A). The expression of these four cysteine substitution mutants were determined by Western blot analyses (Fig. 5B).

To clarify the mechanism underlying these results, we compared the protein structures between the full length of the CAP domain and the four individually replaced cysteine mutants predicted by I-TASSER. The replacement of cysteine with serine was predicted to change the protein structures. For example, the native CAP domain could form five α-helixes and four β-strands, but the CAP^C154S^ mutant could form another α-helix, the CAP^C238S^ mutant lacked a part of the β-strand, and the CAP^C259S^ mutant could also form another α-helix but lacked a part of the β-strand, as shown in the blue dotted boxes in Fig. S3 in the supplemental material.

10.1128/mSphere.00883-20.3 FIG S3 Predicted structure of CAP domain and CAP mutants. I-TASSER was used to predict the structure of CAP domain and its mutants to find out the structural differences. The obvious differences between CAP domain and its mutants are indicated in blue dashed boxes, and the cysteine residues or disulfide bridges are indicated in the green belt. Download FIG S3, TIF file, 1.0 MB. Copyright © 2021 Han et al. 2021 Han et al. https://creativecommons.org/licenses/by/4.0/ This content is distributed under the terms of the Creative Commons Attribution 4.0 International license.

Next, we calculated the structure variations between the native CAP domain and the cysteine substitution mutants with the root-mean-square deviation (RMSD) of the atomic position value. The results revealed that the CAP^C243S^ mutant showed the lowest RMSD value compared to that of the CAP^C154S^, CAP^C238S^, and CAP^C259S^ mutants, indicating only a minor structural variation of the CAP domain when the C^243^ residue was replaced with serine.

These results suggested that cysteine residues C^154^, C^238^, and C^259^ are required for CcCAP1’s activity in suppressing cell death.

### CcCAP1 localizes to the cytoplasm and the nucleus in *N. benthamiana* leaves.

**FIG 6:**
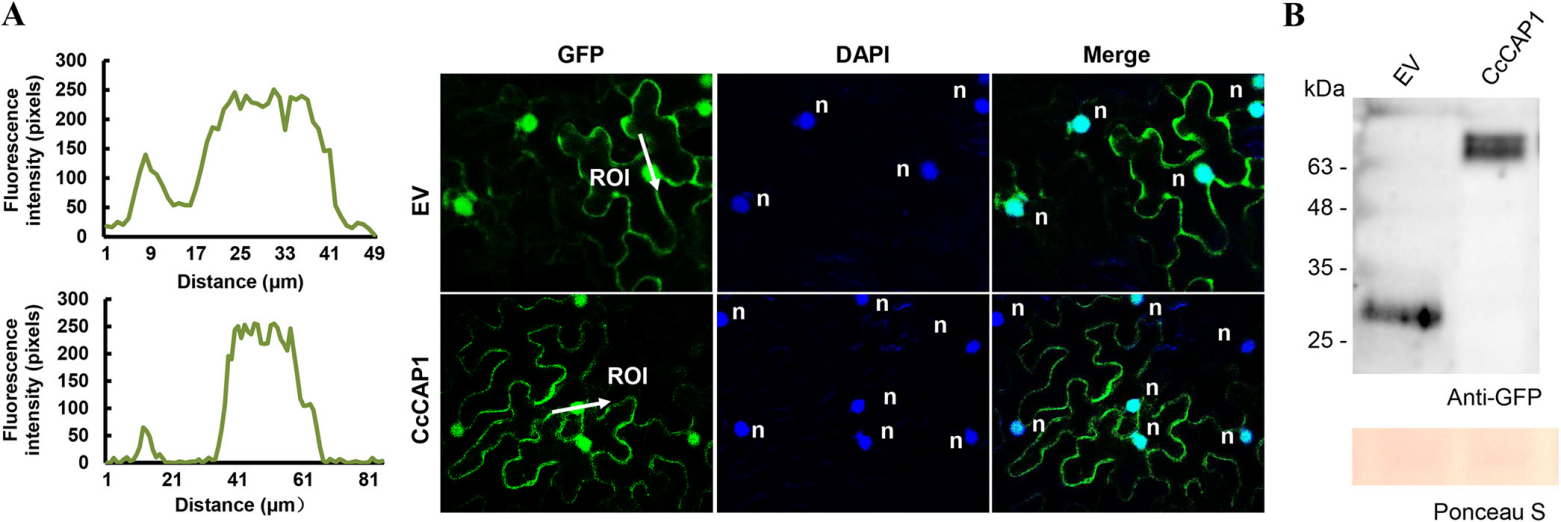
Localization of CcCAP1 in both the nucleus and cytoplasm in N. benthamiana. (A) Subcellular localization was observed 3 h after nucleus being stained with DAPI at 2 dpa of CcCAP1-pBinGFP2. n, nucleus. The white arrow indicates the region of interest, and the line chart indicates the fluorescence intensity of the region of interest. (B) Western blot analysis of proteins in *N. benthamiana* transiently expressing GFP control and CcCAP1 fused with an N-terminal GFP.

To determine the subcellular localization of CcCAP1, we transiently expressed N-terminal GFP-tagged CcCAP1 (without a signal peptide) in N. benthamiana leaves via the *Agrobacterium* infiltration assay. Confocal microscopy showed that the fluorescence of the GFP-tagged CcCAP1 protein was present in both the nucleus and cytoplasm of N. benthamiana at 2 days postagroinfiltration (dpa), which was similar to the localization of the GFP control, and the nuclear localization was verified by DAPI (4′,6′-diamidino-2-phenylindole) staining (Fig. 6A). To support this result, we reconstructed another three constructs tagged with C-terminal GFP expressing the full-length CcCAP1 (SP-CcCAP1), CcCAP1 without a signal peptide (CcCAP1), and CcCAP1 with a signal peptide of plant PR-1 (PR1SP-CcCAP1). All of these proteins localized to the cytoplasmic and nuclear space in N. benthamiana, as shown in Fig. S4. The expression of the GFP control and GFP-fused CcCAP1 in subcellular localization assays was determined by Western blot analyses (Fig. 6B). The results suggested that CcCAP1 localized to the cytoplasm and the nuclei in N. benthamiana leaves.

10.1128/mSphere.00883-20.4 FIG S4 Localization of C-terminal GFP-tagged CcCAP1 in N. benthamiana. (A) Subcellular localization was observed 3 h after nucleus being stained with DAPI at 2 dpa of CcCAP1-pZYGC. n, nucleus. The white arrow indicates the region of interest, and the line chart indicates the fluorescence intensity of the interest region. (B) Western blot analysis of proteins in N. benthamiana transiently expressing GFP control and different types of CcCAP1 fused with a C-terminal GFP tag. Download FIG S4, TIF file, 2.7 MB. Copyright © 2021 Han et al. 2021 Han et al. https://creativecommons.org/licenses/by/4.0/ This content is distributed under the terms of the Creative Commons Attribution 4.0 International license.

### Transient expression of CcCAP1 inhibits the immune responses of *N. benthamiana* and promotes the infection of *Botrytis cinerea*.

**FIG 7:**
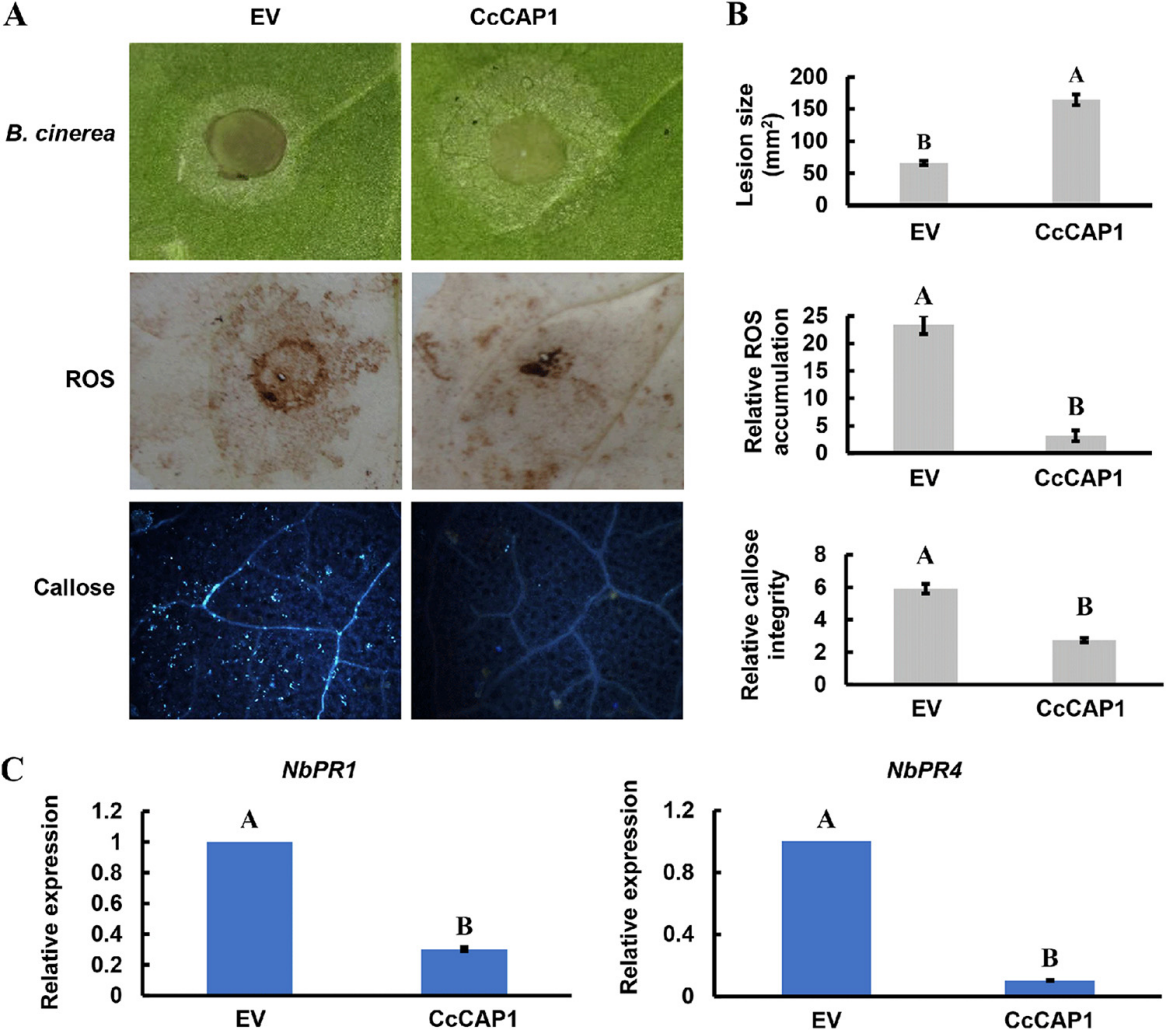
Suppression of the immune responses and enhancement of susceptibility to pathogen of N. benthamiana by overexpression of CcCAP1. (A) Representative infection symptoms, ROS accumulation, and callose deposition on leaves of CcCAP1-pBinGFP2 or EV agroinfiltrated N. benthamiana at 2 dpi with *B. cinerea*. (B) Quantification of lesion area, ROS, and callose intensity with ImageJ. (C) Transcriptional levels of defense-related genes were detected at 2 dpi with *B. cinerea* on leaves of CcCAP1-pBinGFP2 or EV agroinfiltrated N. benthamiana. This experiment was performed three times with similar results. Each assay was performed on at least six independent biological repeats. The statistical analyses were conducted by SPSS v16.0, which was used to analyze the experimental data, and Duncan’s test at *P* = 0.05 was used to determine the differences in the expression level of defense-related genes. Bars indicate ± SE. The letters above the error bars indicate the different groups with statistical significance (*P ≤ *0.01).

As described above, CcCAP1 is essential for fungal virulence and suppressed the PCD triggered by BAX and INF1. Thus, CcCAP1 might have the potential to modulate the immunity of N. benthamiana. To test this, we agroinfiltrated different sides of N. benthamiana leaves with the CcCAP1-pBinGFP2 plasmid or the empty vector (EV) control. At 1 dpa, we inoculated the N. benthamiana leaves with mycelia plugs of B. cinerea, a notorious necrotrophic pathogen that causes visible disease symptoms on N. benthamiana (79). As expected, at 2 dpi with *B. cinerea*, we observed obvious disease symptoms and larger lesion size in leaves expressing CcCAP1 compared to that on leaves expressing the EV control (Fig. 7A and B). The results indicated that CcCAP1 could manipulate N. benthamiana immunity to promote the infection of *B. cinerea*.

ROS production, callose accumulation, and induced expression of defense-related genes are important plant immune responses against pathogens. To verify whether CcCAP1 regulates plant immunity via the responses listed above, we assessed the ROS accumulation, callose deposition, and expression of defense-related genes in the N. benthamiana plants described above at 2 dpi with *B. cinerea*. As shown in Fig. 7A and B, ROS accumulation and callose deposition in leaves expressing CcCAP1 were significantly lower than those in leaves expressing the EV control, and the expression of two defense-related marker genes, *NbPR1* from the salicylic acid signaling pathway and *NbPR4* from the jasmonic acid signaling pathway, was significantly suppressed in leaves expressing CcCAP1 compared to leaves expressing the EV control (Fig. 7C). These results suggested that the expression of CcCAP1 in N. benthamiana inhibited plant immune responses.

### Nuclear localization is essential and sufficient for the immune-inhibiting activity of CcCAP1.

**FIG 8:**
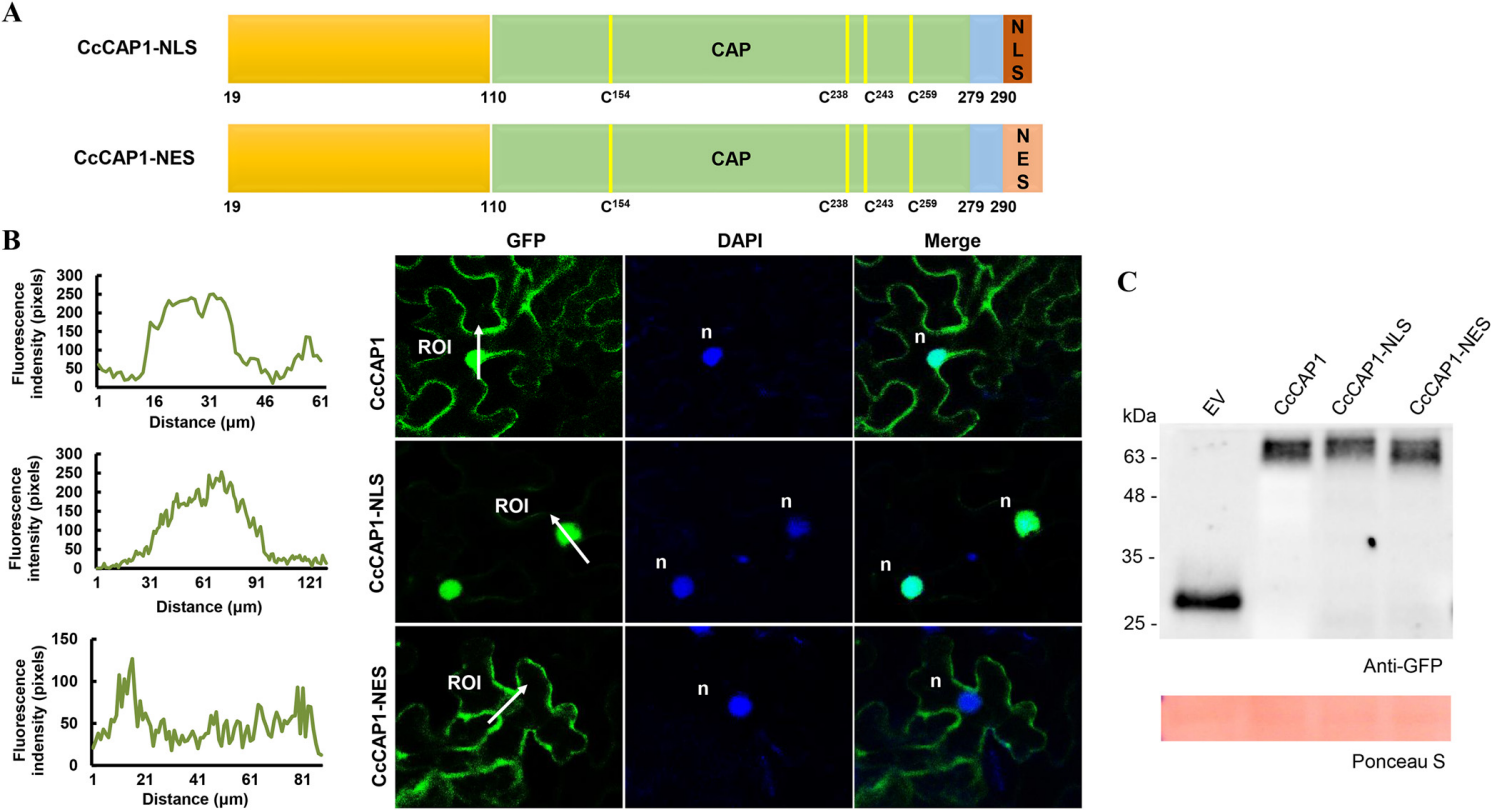
Artificial alteration of the subcellular localization of CcCAP1 with NLS and NES sequence. (A) Schematic diagram of CcCAP1 that was artificially added with NLS or NES sequence at the C terminus. NLS, nuclear localization signal, indicated in brown; NES, nuclear export signal, indicated in pink. (B) Confocal microscopy images showing the subcellular localization of CcCAP1 and its modified mutants. Alteration of the subcellular localization of CcCAP1 was observed at 2 dpa, with the nucleus being stained with DAPI 3 h before confocal observation. n, nucleus. (C) Western blot analysis of proteins in *N. benthamiana* transiently expressing GFP control and GFP-tagged CcCAP1, CcCAP1-NLS, and CcCAP1-NES.

As described above, CcCAP1 localized to both the nucleus and cytoplasm, and it could inhibit plant immune responses. To estimate which subcellular localization of CcCAP1 was required for its plant immune-inhibiting activity, we generated two additional constructs, CcCAP1-NLS-pBinGFP2 and CcCAP1-NES-pBinGFP2, by artificially adding a nuclear localization signal (NLS) sequence or a nuclear export signal (NES) sequence to the C terminus of CcCAP1, which could specifically bring CcCAP1 to the nucleus or export CcCAP1 out of the nucleus (Fig. 8A). Confocal microscopy observations showed that the fluorescence of CcCAP1-NLS almost exclusively concentrated in the nucleus, while the fluorescence intensity of CcCAP1-NES was dramatically reduced in the nucleus compared to that of CcCAP1 (Fig. 8B), indicating that CcCAP1-NES proteins were mostly exported out of the nucleus. The expression of GFP and GFP fusion proteins was determined by Western blotting (Fig. 8C).

**FIG 9:**
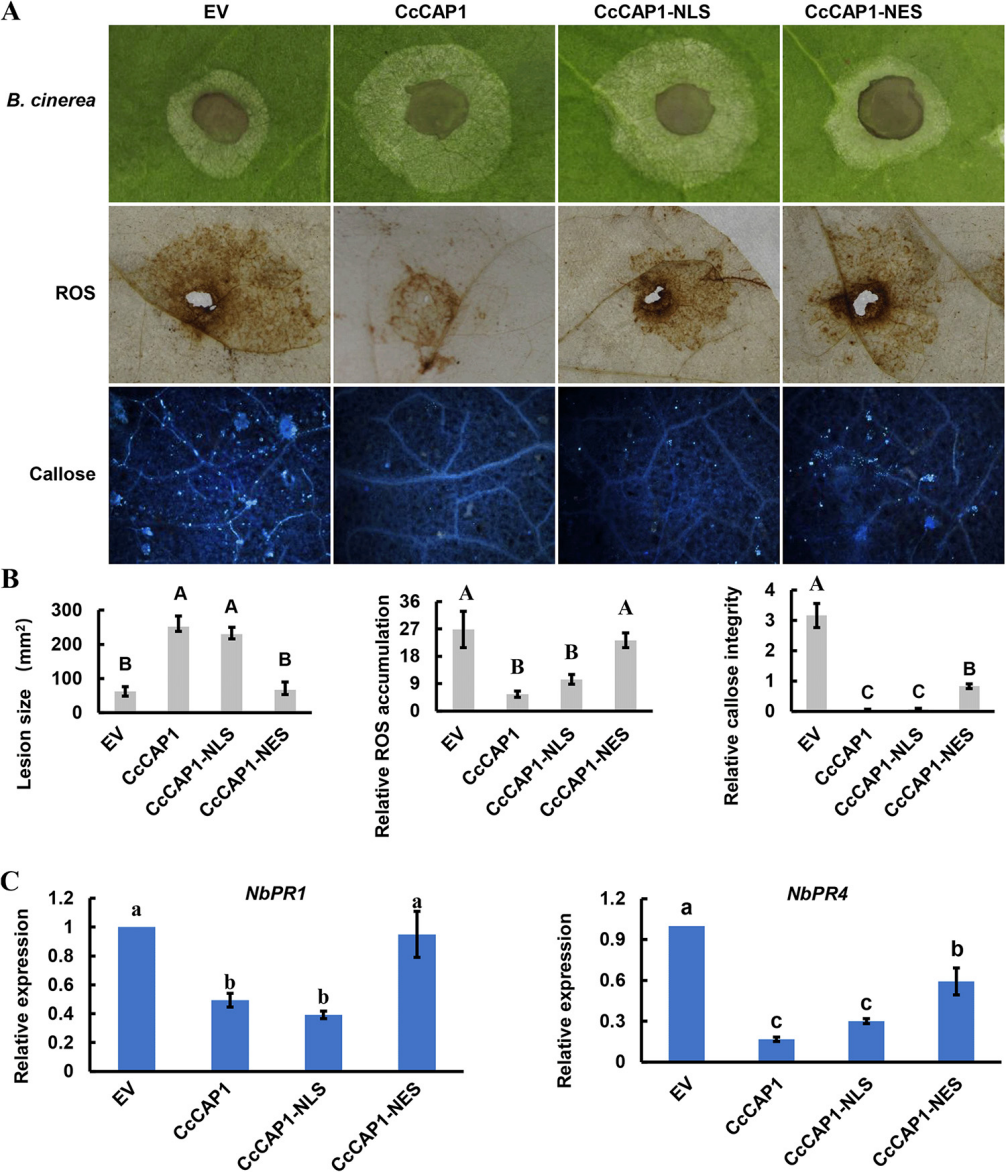
Significant role of the nuclear localization in immunoinhibiting activity of CcCAP1. (A) Representative infection symptoms, ROS accumulation, and callose deposition on leaves of EV, CcCAP1-pBinGFP2, CcCAP1-NLS-pBinGFP2, and CcCAP1-NES-pBinGFP2 agroinfiltrated N. benthamiana at 2 dpi with *B. cinerea*. (B) Quantification of lesion area, ROS, and callose intensity with ImageJ. (C) Relative transcriptional levels of defense-related genes were detected at 2 dpi with *B. cinerea* on leaves of EV, CcCAP1-pBinGFP2, CcCAP1-NLS-pBinGFP2, or CcCAP1-NES-pBinGFP2 agroinfiltrated N. benthamiana. This experiment was performed three times with similar results. Each assay was performed on at least six independent biological repeats. SPSS v16.0 was used to analyze the experimental data, and Duncan’s test at *P* = 0.05 was used to determine the differences. Bars indicate ± SE. The letters above the error bars indicate the different groups with statistical significance (*P* ≤ 0.01, *P* ≤ 0.05).

To determine whether the altered localization of CcCAP1 affect its immune-inhibiting functions, we inoculated the leaves of N. benthamiana transiently expressing the EV control, CcCAP1, CcCAP1-NLS, or CcCAP1-NES with *B. cinerea* mycelial plugs. The leaves expressing CcCAP1 and CcCAP1-NLS developed significantly larger infection lesions compared to the leaves expressing CcCAP1-NES and the EV control (Fig. 9A and B). Also, there was no obvious difference between the lesion sizes of the leaves expressing CcCAP1-NES and the EV. This suggested that the nuclear localization of CcCAP1 was required for promoting the infection of *B. cinerea*.

In addition, we calculated the changes of plant immune responses corresponding to the different localization of CcCAP1 in N. benthamiana. As shown in Fig. 9A and B, the leaves expressing CcCAP1 and CcCAP1-NLS showed similar levels of ROS accumulation and callose deposition after inoculation with *B. cinerea*, while these levels were significantly higher in the leaves expressing the EV and CcCAP1-NES, indicating that the export of CcCAP1 from the nucleus almost completely lost the ability to suppress the ROS accumulation and callose deposition in the plant. Furthermore, the expression of the defense-related marker genes *NbPR1* and *NbPR4* in leaves expressing CcCAP1 and CcCAP1-NLS was significantly reduced compared to that in the leaves expressing the EV and CcCAP1-NES (Fig. 9C). These results implied that the nuclear localization of CcCAP1 was essential and sufficient for its immune-inhibiting activity.

## DISCUSSION

Phytopathogens secrete lots of effectors to regulate plant immunity and promote infection (80). Generally, the effectors are strongly induced upon infection with the host, and their expression patterns are closely related to the different infection stages. Some effectors are expressed in a stage-, organ-, and host-specific manner and play various roles in plant-microbe interactions (81, 82). In addition, pathogens with different lifestyles develop diverse strategies to subvert plant defenses. *C. chrysosperma*, a necrotrophic plant pathogen, tends to quickly kill the plant cell to extract nutrients. However, it is believed that necrotrophic plant pathogens, such as *B. cinerea* and Sclerotinia sclerotiorum, might experience a brief biotrophic phase before killing the plant cell to overcome the plant defenses (83–87).

In this study, we selected and functionally characterized a small, cysteine-rich protein, CcCAP1, which belongs to the CAP superfamily, from the poplar canker fungus *C. chrysosperma* (Fig. 1). CcCAP1 was strongly induced during the early stages of infection. The results showed that CcCAP1 was essential for the virulence of the fungus. It could inhibit the plant PCD induced by BAX and INF1 and modulated the plant defense responses. Further analysis revealed that nuclear localization of CcCAP1 was essential and sufficient for its immune suppression activities. These results suggested that CcCAP1 is an important virulence-related effector in *C. chrysosperma*.

Pathogens employ different effectors during the different stages of infection, which may play specific functions, such as the “immediate early effectors” or “early effectors” as described previously (88). The expression of CcCAP1 was significantly upregulated during the early infection stage in poplar, with the maximal expression levels at 2 dpi (∼7-fold versus the control), and then dramatically declined to a low expression levels during 3 to 12 dpi, at which point its expression was similar to the expression levels of CcCAP1 in the vegetative growth stage. These results indicated that CcCAP1 acts as an important virulence factor that is involved in the initial invasion and colonization of *C. chrysosperma* in its host. Therefore, it is possible that CcCAP1 mainly participates in the brief biotrophic phase of infection.

Here, we found that expression of CcCAP1 could suppress the PCD elicited by BAX and INF1. INF1, a well-known PAMP from oomycetes, is an important signaling component of PTI, indicating that CcCAP1 could inhibit the PTI-related PCD in the host, which might help *C. chrysosperma* avoid being recognized by the host. In addition, the typical PTI responses, including ROS accumulation, callose deposition, and the induced expression of defense-related genes, were also compromised by transient expression of CcCAP1 in N. benthamiana. Many effectors from different pathogens have been shown to inhibit the PTI-associated response (43, 89), and several molecular mechanisms underlying the suppression of PTI responses have been characterized. For example, the PAMP Flg22 from bacteria can quickly elicit the PTI responses of the host by quickly activating the MAPK signaling pathway, while the subsequent effectors could inactivate the MAPK signaling pathway (90, 91). Therefore, further research is needed to determine the signaling network underlying the activity of CcCAP1 in the suppression of PTI responses.

The CAP proteins constitute a large protein superfamily with members found in all kingdoms of life (61, 62). Functional analysis of the secreted CAP members pathogenesis-related proteins PRY1 and PRY2 in Saccharomyces cerevisiae showed that they are involved in sterol binding and the export of acetylated cholesterol and, more importantly, that the CAP domain alone was sufficient for their functions (63, 64). In this study, we found that transient expression of CcCAP1 in N. benthamiana could suppress the PCD triggered by INF1, and expression of the CAP domain alone could also suppress the PCD triggered by INF1, indicating that the CAP domain is sufficient for its functions. In addition, the highly conserved sequence of the CAP domain may suggest similar functions among the CAP proteins of different organisms. For example, the defects of pathogenesis-related proteins (PRY) mutants in S. cerevisiae could be restored by the human CAP protein cysteine-rich secretory protein 2 (CRISP2) (63).

Here, we found six CAP homologs of CcCAP1 in the PHI-base database, including Fvscp1 from F. verticillioides, Rbt4 from C. albicans, Gr-VAP1 from *G. rostochiensis*, and FgPr-1l-2, FgPr-1l-3, and FgPr-1l-4 from F. graminearum. Importantly, four of these (Fvscp1, Rbt4, Gr-vap1, and FgPr-1l-4) were found to positively regulate the pathogen virulence (Fig. 1), and the CcCAP1 deletion mutants were also significantly reduced in fungal virulence, indicating that the CAP members function in pathogen virulence. The observation that CAP member mutants do not affect pathogenicity may be due to partially redundant roles in virulence as described in C. albicans (65).

Remarkably, the CcCAP1 homolog Gr-VAP1 (small, with a signal peptide, and rich in cysteines) targets the papain-like cysteine protease Rcr3 and is recognized by the Cf-2 receptor (an extracellular plant immune receptor protein), resulting in defense-related PCD in tomato (*Solanum pimpinellifolium*) (68). A similar result was found in the *Cladosporium fulvum* effector Avr2, which interacts with tomato Rcr3 and activates Cf-2 function in immune signaling cascades, thus resulting in effector-triggered immunity (92). However, the interaction of CcCAP1 and putative targets is unknown and needs to be elucidated in the future, which will help us to better understand the functions of CcCAP1.

Many studies have reported that effectors localize to specific subcellular compartments to achieve their functions, and the localization of effectors may correspond to their colocalized plant targets (31, 49, 50, 54–57). PsAvh52 (containing a potential NLS sequence) from *P. sojae* localizes to the plant nucleus to enhance plant susceptibility by relocating a host cytoplasmic transacetylase, GmTAP1, into nuclear speckles; when the localization of PsAvh52 is artificially altered, the plant target protein GmTAP1 would not transfer to the nucleus (58). Another RxLR effector, Avh241 from *P. sojae*, localizes to the plasma membrane to induce plant cell death, and deletion mutants of different regions of Avh241 altered its localization and subverted the ability to trigger cell death (59). In addition, a Ralstonia solanacearum effector, RipAB, and a *Verticillium dahlia* effector, VdSCP7, both required an NLS to trigger cell death in N. benthamiana (93, 94). Some effectors localize to the host nucleus and target the host transcription factors or RNA interference components to impair plant defenses (37, 95, 96). An RxLR effector, Avh238^P6497^, localizes to both the nucleus and cytoplasm, and Avh238^P6497^ could trigger plant cell death and suppress plant cell death elicited by INF1 in N. benthamiana (88, 97). Further analysis revealed that the nuclear localization of Avh238 was required for the induction of cell death, but the cytoplasmic localization of Avh238 was required for the suppression of INF1-triggerred cell death (97).

**FIG 10:**
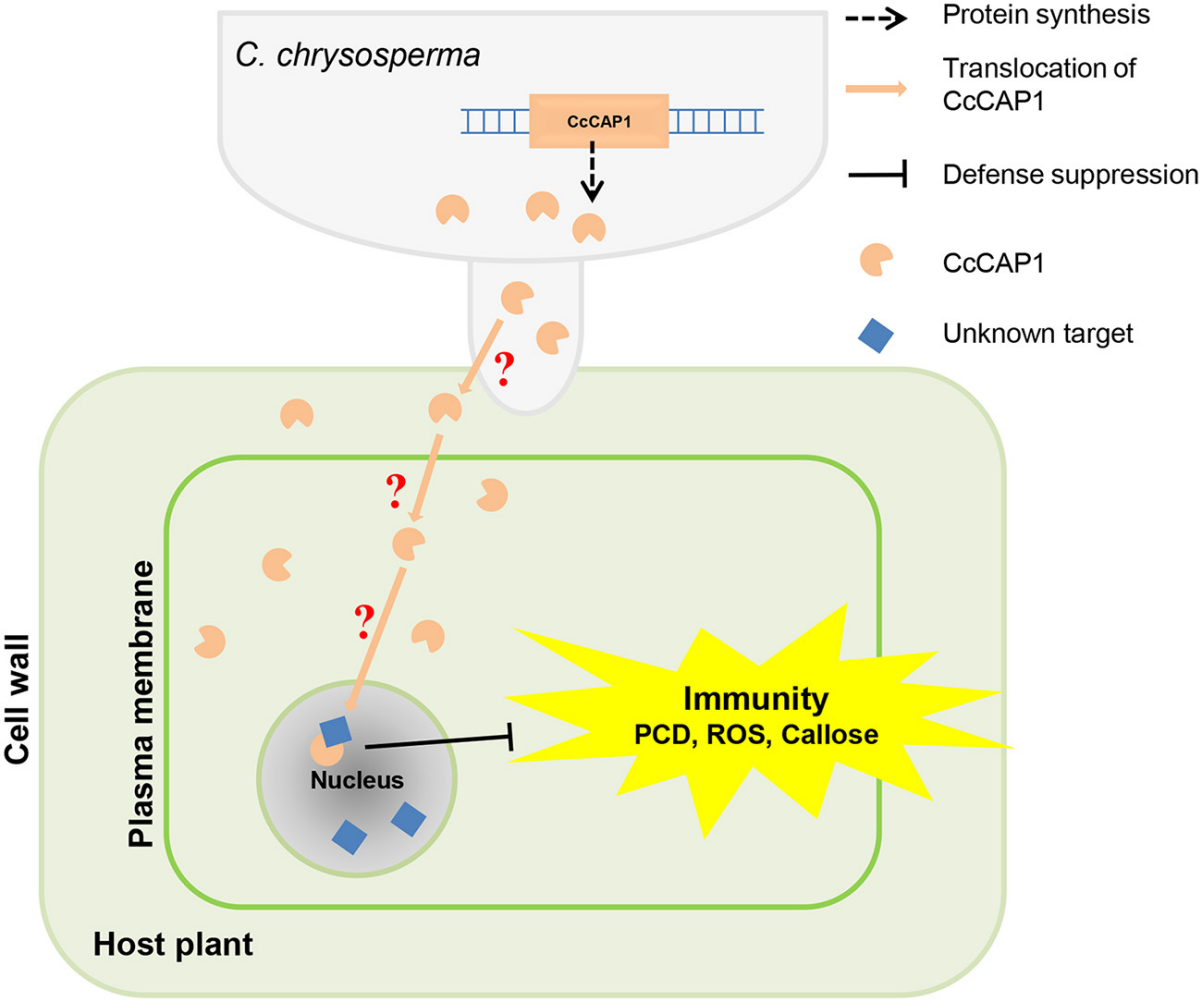
Hypothesis of CcCAP1 functions during *C. chrysosperma*-host interaction. Inductively expressed CcCAP1 proteins were first transported to the apoplastic space; they then translocate to the cytoplasmic space and finally to the nucleus to inhibit the plant immunity.

Our results showed that CcCAP1 localizes to both the plant nucleus and cytoplasm (Fig. 6 and see Fig. S4). Artificial alteration of the localization of CcCAP1 by adding an NLS or NES sequence to the C terminus of CcCAP1 GFP-tagged proteins showed that it was the nuclear localization rather than the cytoplasmic localization of CcCAP1 that is essential and sufficient for its suppression activity of the PTI response, including the ROS accumulation, callose deposition, and the expression of defense-related genes in N. benthamiana (Fig. 7 to 9). However, the function of cytoplasmic CcCAP1 is still unclear and requires further analysis. Furthermore, we also found that both the full-length CcCAP1 (without a signal peptide) and the CAP domain alone were autoactivated (data not shown), indicating that CcCAP1 may act as a transcriptional regulator that manipulates the expression of downstream genes and thus interferes with plant immunity by entering the plant nucleus. These results suggest that CcCAP1 may target nucleus-localized host proteins to regulate plant immunity, as depicted in Fig. 10.

In conclusion, in this study we identified a virulence-related effector, CcCAP1, in *C. chrysosperma* that is conserved in different fungi as a CAP superfamily member. It could suppress BAX- and INF1-induced cell death, resulting in the enhanced susceptibility to *B. cinerea* and subverted immune responses in N. benthamiana. The CAP domain and cysteine residues are required for the activity of CcCAP1 in suppressing PTI-induced PCD, and the plant nuclear localization of CcCAP1 was essential for its function. These results suggested that CcCAP1, a virulence CAP member, was employed by *C. chrysosperma* to modulate the plant immunity in N. benthamiana.

## MATERIALS AND METHODS

### Bioinformatics analysis.

For screening of candidate effector genes in the whole-genome sequence of *Cytospora chrysosperma*, which had been sequenced by our lab (unpublished data), we used the SignalP 4.0 server (http://www.cbs.dtu.dk/services/SignalP-4.0/) (98), the TMHMM server v.2.0 (http://www.cbs.dtu.dk/services/TMHMM/) (99), the TargetP 1.1 server (http://www.cbs.dtu.dk/services/TargetP/) (100), Interpro (http://www.ebi.ac.uk/interpro/), and the PHIB BLAST (http://phi-blast.phi-base.org/) online websites, as well as the NCBI database (https://www.ncbi.nlm.nih.gov/). We used default parameters to predict a signal peptide sequence, other transmembrane domains, the potential localization, the functional domain, the homologous proteins that were already investigated, and CAP superfamily members from selected species, respectively. In addition, we used Mega 6.0, Clustalx, and BioEdit Sequence Alignment Editor for multiple sequence alignment. Mega 6.0 was also used for phylogenetic tree construction. The I-TASSER online server (https://zhanglab.ccmb.med.umich.edu/I-TASSER/) was used for prediction of the protein structure. The models were displayed using the software PyMOL.

### Strains and plant growth conditions.

The *C. chrysosperma* wild-type (WT) strain (CFCC 89981), isolated from *Populus beijingensis*, was preserved in the forest pathology laboratory of Beijing Forestry University (strain G-YS-11-C1) (101). The necrotrophic pathogen *Botrytis cinerea* was kindly provided by associate professor Dai Tingting of Nanjing Forestry University. The *C. chrysosperma* WT strain, deletion mutants, and complementation mutants, as well as the *B. cinerea* WT strain, were generally grown and maintained on PDA medium at 25°C in the dark. The Agrobacterium tumefaciens strain GV3101 (pJIC SA_Rep), provided by associate researcher Tingli Liu of the Nanjing Academy of Agricultural Sciences, was used for agroinfiltration of Nicotiana benthamiana. N. benthamiana was grown in a greenhouse at 25°C and 70% relative humidity with a 16-h/8-h day/night photoperiod. The materials used for the pathogenicity test were selected from the healthy annual branches of the susceptible species *Populus euramericana* and cultured at 25°C in the dark.

### Construction of deletion and complementation mutants.

The full-length open reading frame sequence of CcCAP1 was knocked out using a split-marker method combined with PEG-mediated protoplast transformation, as described previously (102). According to this method, the upstream (∼1.2 kb) and downstream (∼1.2 kb) flanking sequences of CcCAP1 were amplified by primer pairs CcCAP1-5Ffor/CcCAP1-5Frev and CcCAP1-3Ffor/CcCAP1-3Frev, respectively. The hygromycin B resistance cassette, including ∼20 bp of overlap sequence with the 5′ and 3′ flanking sequences, was amplified by the primer pair hygromycinfor and hygromycinrev. The resulting upstream and downstream fragments were fused with two-thirds of the hygromycin B resistance cassette by overlap PCR with primer pairs CcCAP1-5Ffor/HY-R and YG-F/CcCAP1-3Frev, respectively. The two overlapping fragments were directly transformed into the protoplasts of the *C. chrysosperma* WT strain, and the transformants were selected using the primer pairs External-CcCAP1for/External-CcCAP1-rev and Internal-CcCAP1for/Internal-CcCAP1rev, respectively. To analyze the homologous recombination events in the transformants, Southern blotting was conducted with a DIG High Prime DNA labeling and detection starter kit I according to the manufacturer’s protocol (Roche, Germany). Smal was used to digest the genomic DNA extracted from the WT strain and the transformants. The probes were amplified by the primers Probe CcCAP1for and Probe CcCAP1rev from *C. chrysosperma* and labeled with DIG primer.

For generation of the CcCAP1 gene complementation construct, the whole CcCAP1 gene cassette containing an upstream ∼1.5-kb native promoter sequence, full-length open reading frame, and a downstream ∼0.2-kb terminator sequence was cloned from gDNA using the primer pair CcCAP1-Compfor/CcCAP1-Comprev. The resulting PCR products were cotransformed with a Geneticin-resistant cassette into protoplasts of the ΔCcCAP1-8 strains, and the transformants were selected on PDA medium supplemented with 25 μg/ml hygromycin and 50 μg/ml Geneticin. Successful complementation was confirmed by PCR with the primer pair Internal-CcCAP1for/Internal-CcCAP1rev, and the complementation strain was named ΔCcCAP1/C in this study.

### Plasmid construction.

To determine the cell death-inducing or -inhibiting activity, the CcCAP1 coding sequence without the signal peptide (the mature type) was amplified from the *C. chrysosperma* cDNA library with gene-specific primer pairs CcCAP1-pGR106for/CcCAP1-pGR106rev. The amplicons were then cloned into the PVX vector (pGR106) (103) and digested with specific restriction enzymes (ClaI and SmaI; TaKaRa) to create CcCAP1-pGR106. The pGR106 EV and the following GFP-pGR106, BAX-pGR106, and pBinGFP2 EVs were kindly offered by Daolong Dou from Nanjing Agricultural University.

To test the activity of the CAP domain, the truncated CcCAP1 motifs L, L-C, C, and C-T were cloned from CcCAP1-pGR106 with the primer pairs CcCAP1-L-pGR106for/CcCAP1-L-pGR106rev, CcCAP1-L-C-pGR106for/CcCAP1-L-C-pGR106rev, CcCAP1-C-pGR106for/CcCAP1-C-pGR106rev, and CcCAP1-C-T-pGR106for/CcCAP1-C-T-pGR106rev, respectively, and ligated into the pGR106 vector digested by ClaI and SmaI to generate CcCAP1-L-pGR106, CcCAP1-L-C-pGR106, CcCAP1-C-pGR106, and CcCAP1-C-T-pGR106, respectively.

For cysteine analysis, the single point mutation method was adopted to generate the CAP mutants CcCAP1-C^C154S^-pGR106, CcCAP1-C^C238S^-pGR106, CcCAP1-C^C243S^-pGR106, and CcCAP1-C^C259S^-pGR106 by substitution of cysteine with serine using a Fast Site-directed mutagenesis kit (Tiangen) with CcCAP1-C-pGR106 as the template.

To investigate the localization of CcCAP1 in N. benthamiana, the pBinGFP2 plasmid (59) was applied to generate CcCAP1-pBinGFP2 using the same method as that used for CcCAP1-pGR106 with different primer pairs (CcCAP1-pBinGFP2for/CcCAP1-pGR106rev) and different restriction enzymes (KpnI and SmaI). For construction of C-terminal GFP fusion, the full length of CcCAP1 (SP-CcCAP1), CcCAP1 without its native signal peptide (CcCAP1) and CcCAP1 with a signal peptide from plant PR-1 (PR1SP-CcCAP1) were introduced into pZYGC plasmid digested with KpnI and BamHI. These fragments were cloned with primer pairs SP-CcCAP1-pZYGCfor/SP-CcCAP1-pZYGCrev, CcCAP1-pZYGCfor/CcCAP1-pZYGCrev, and PR1SP-CcCAP1-pZYGCfor/PR1SP-CcCAP1-pZYGCrev. The pZYGC plasmid was kindly offered by Ningjia He from Xinan University.

To determine the active subcellular site of CcCAP1, nuclear localization signal (NLS) or nuclear export signal (NES) sequences were added to the C terminus of CcCAP1 using the primer pairs CcCAP1-pBinGFP2for/CcCAP1-NLS-pBinGFP2rev and CcCAP1-pBinGFP2for/CcCAP1-NES-pBinG-FP2rev, respectively, with CcCAP1-pBinGFP2 used as the template and the same restriction enzymes, to create the CcCAP1-NLS-pBinGFP2 and CcCAP1-NES-pBinGFP2 constructs. All constructs were validated by sequencing by Thermo Fisher, Beijing, China.

### Transient expression mediated by *Agrobacterium* infiltration.

For heterologous expression of the above constructs, we used *Agrobacterium*-based methods as described previously, which are widely used in *Solanum* plants (104, 105). The constructs were chemically transformed into *Agrobacterium* strain GV3101, and the cells were cultivated in Luria-Bertani medium at 28°C in a shaking incubator at 200 rpm for 48 h. The bacteria were then pelleted by centrifugation and resuspended in MgCl_2_ buffer (10 mM MgCl_2_, 10 mM MES, and 200 μM acetosyringone) in the dark for 3 h at 28°C before infiltration, as described previously (94). For infiltration, suspended Agrobacterium cells were adjusted to a final optical density at 600 nm of 0.4, and the cell suspension was infiltrated into plant leaves using a 1-ml syringe without a needle.

To determine the cell death-inducing or -inhibiting activity of the candidate effector protein, the CcCAP1-pGR106 construct and the GFP-pGR106 control construct were agroinfiltrated into the leaves of N. benthamiana 1 day before BAX or INF1 agroinfiltration in the same site, with BAX, INF1, and GFP as controls. Symptom development was monitored visually at 5 dpa. The same methods were used to evaluate the cell death-inhibiting activity of CAP domain and cysteine substitution mutants.

### Protein extraction and Western blots.

Agroinfiltrated N. benthamiana leaves were harvested at 2 dpa and used for total protein extraction with a plant protein extraction kit (BC3720; Solarbio) according to the manufacturer’s instructions.

For the Western blot analysis, total proteins from leaves were separated using SDS-PAGE and transferred to polyvinylidene difluoride membranes. The membranes were then blocked in TBST with 5% nonfat dry milk with gentle shaking at room temperature for 1 h. Specific anti-GFP (catalog no. 2956; CST) or anti-HA (catalog no. 3724; CST) antibodies were added to blocking buffer at a 1:1,000 dilution. Membranes were incubated with antibodies for overnight at 4°C with gentle shaking. Subsequently, the membranes were washed three times and then incubated with horseradish peroxidase-labeled goat anti-rabbit IgG(H+L) secondary antibodies (catalog no. 111-035-003, Jackson) at a 1:15,000 dilution. Protein bands were detected using an ECL Western blot kit (PE0010; Solarbio).

### Confocal microscopy analysis.

The N. benthamiana leaves agroinfiltrated with CcCAP1-pBinGFP2, CcCAP1-NLS-pBinGFP2, CcCAP1-NES-pBinGFP2, and the EV control were stained with 5 μg/ml DAPI at 2 dpa. Three hours after staining, the leaves were cut into 8 × 8-mm^2^ pieces and mounted in water on glass slides for confocal microscopy analysis. The fluorescence was imaged using a TCS SP8 confocal microscope system (Leica, Germany). The excitation wavelengths were 488 nm for GFP and 405 nm for DAPI.

### Infection assay.

For the pathogenicity test, 15-cm-long healthy annual branches of the susceptible species *Populus euramericana* were selected and scalded with a 5-mm-diameter hot iron bar to be inoculated with 5-mm-diameter *C. chrysosperma* mycelial plugs. After inoculation, the twigs were sealed with sealing film and placed in trays with distilled water to maintain humidity and then incubated at 25°C in the dark. In the next several days, the twigs were sprayed with water to maintain moisture for pathogen infection. Lesions were photographed and measured at 4 dpi.

To identify the immunoregulation activity and subcellular site of action of CcCAP1, the detached N. benthamiana leaves were inoculated with 5-mm-diameter *B. cinerea* mycelial plugs at the injection site at 1 dpa with the EV control, CCG-07874-pBinGFP2, CcCAP1-NLS-pBinGFP2, and CcCAP1-NES-pBinGFP2. Lesion symptoms were photographed and measured at 2 dpi, followed by 3,3′-diaminobenzidine (DAB) staining, aniline blue staining, and reverse transcription-quantitative PCR (RT-qPCR) analysis.

### DAB staining.

At 2 dpi with *B. cinerea* after the transient expression of the EV control, CCG-07874-pBinGFP2, CcCAP1-NLS-pBinGFP2, and CcCAP1-NES-pBinGFP2, leaf samples were collected, and leaf segments with an infiltrated area were cut and stained in a freshly made DAB (Sigma)-HCl solution (1 mg/ml [pH 3.8]). The preparation of a DAB staining solution and the staining process followed the procedure described by Thordal-Christensen (106). The stained leaf tissue was cleared of chlorophyll by placing it in a conical flask with 20 ml of 75% ethanol solution, followed by incubation overnight at 37°C. The cleared leaves were photographed with a digital camera.

### Aniline blue staining.

Callose deposition was visualized using the aniline blue staining approach as described previously (107), with some modifications. Infected leaves expressing the EV, CcCAP1-pBinGFP2, CcCAP1-NLS-pBinGFP2, and CcCAP1-NES-pBinGFP2 constructs were soaked in 96% ethyl alcohol at 37°C and 200 rpm overnight. Next, the destained leaves were submerged in 0.05% aniline blue in 0.067 M K_2_HPO_4_ (pH 9.2) at 37°C and 200 rpm overnight and subsequently imaged using a biological microscope.

### RNA extraction and RT-qPCR analysis.

Total RNA was isolated with TRIzol reagent (Invitrogen) and purified with a PureLink RNA minikit (Invitrogen) in accordance with the manufacturer’s instructions. First-strand cDNA was synthesized from 1 μg of RNA with SuperScript IV reverse transcriptase (Invitrogen) according to the manufacturer’s instructions, followed by RT-qPCR with SuperReal Premix Plus (Tiangen, China) using an ABI 7500 real-time PCR system (Applied Biosystems).

For investigation of the CcCAP1 expression levels during *C. chrysosperma* infection on poplar, RNA samples were extracted from twig tissues inoculated with *C. chrysosperma* WT strain at 0, 1, 2, 3, 6, and 12 dpi. The *CcActin* gene in *C. chrysosperma* was used as an internal control to normalize the gene expression of CcCAP1 according to the 2^–ΔΔ^*^CT^* method (108). The experiment was performed in biological triplicate with three independent technical replicates each.

To verify the expression of defense-related genes in N. benthamiana leaves expressing CcCAP1-pBinGFP2, CcCAP1-NLS-pBinGFP2, CcCAP1-NES-pBinGFP2, and the EV, RNA samples were extracted from N. benthamiana leaves inoculated with *B. cinerea* at 2 dpi. The *NbActin* gene in N. benthamiana was used as an internal control. This was performed in biological triplicate with three independent technical replicates each.

All primers used in this study are listed in Table S2 in the supplemental material.

10.1128/mSphere.00883-20.6 TABLE S2 Primers used in this study. Download Table S2, XLSX file, 0.01 MB. Copyright © 2021 Han et al. 2021 Han et al. https://creativecommons.org/licenses/by/4.0/ This content is distributed under the terms of the Creative Commons Attribution 4.0 International license.

### Data analysis.

SPSS v16.0 was used to analyze the experimental data and Duncan’s test at *P* ≤ 0.05 or *P* ≤ 0.01 was used for determining the differences.
