# Supplementary figures and images for: The Cytospora chrysosperma Virulence Effector CcCAP1 Mainly Localizes to the Plant Nucleus To Suppress Plant Immune Responses
Source: mSphere. 2021 Feb 24;6(1):e00883-20. doi: 10.1128/mSphere.00883-20 (PMC8544888; doi:10.1128/mSphere.00883-20)

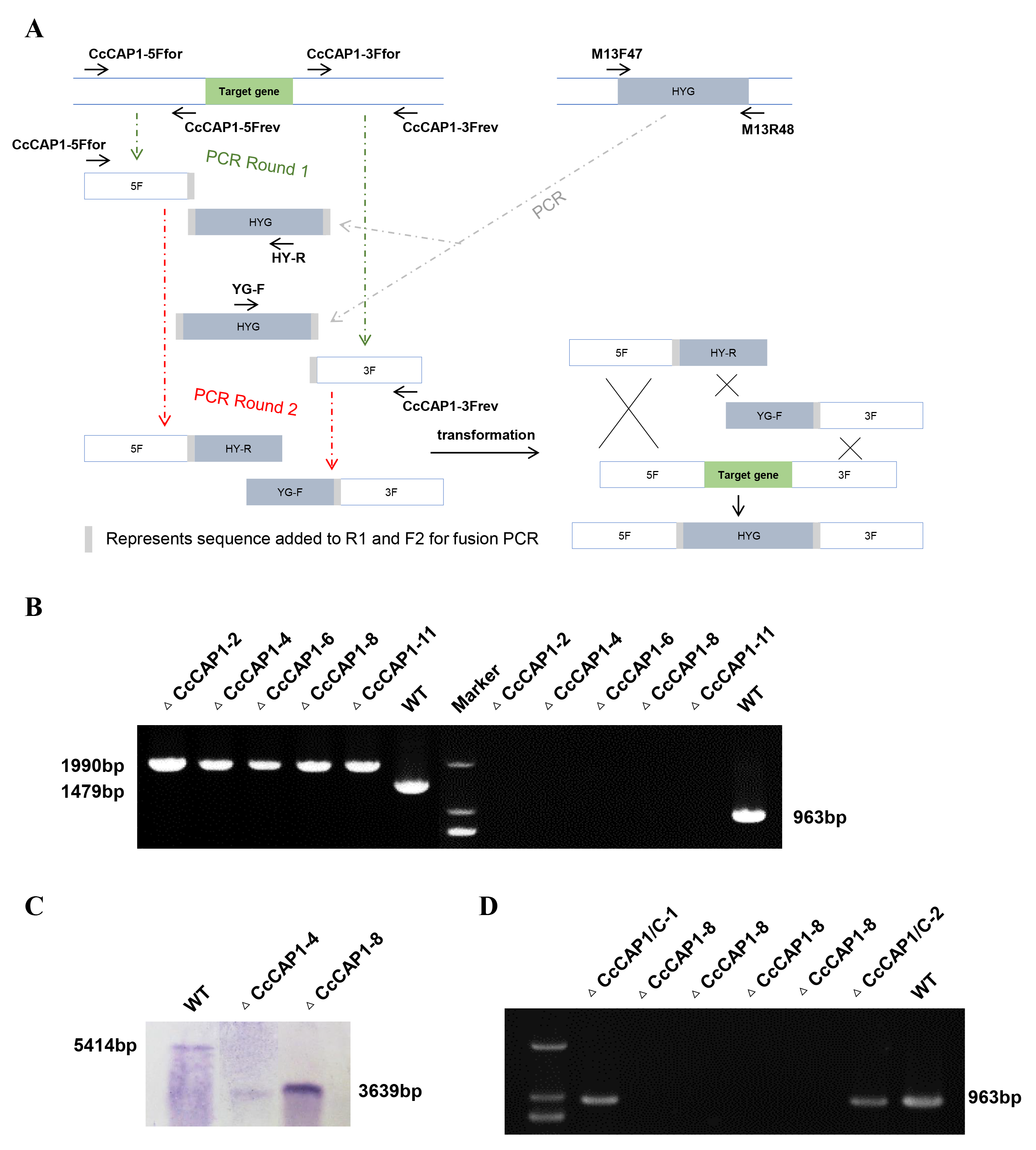

Supplement: FIG S2 [file msphere.00883-20-sf002.tif]

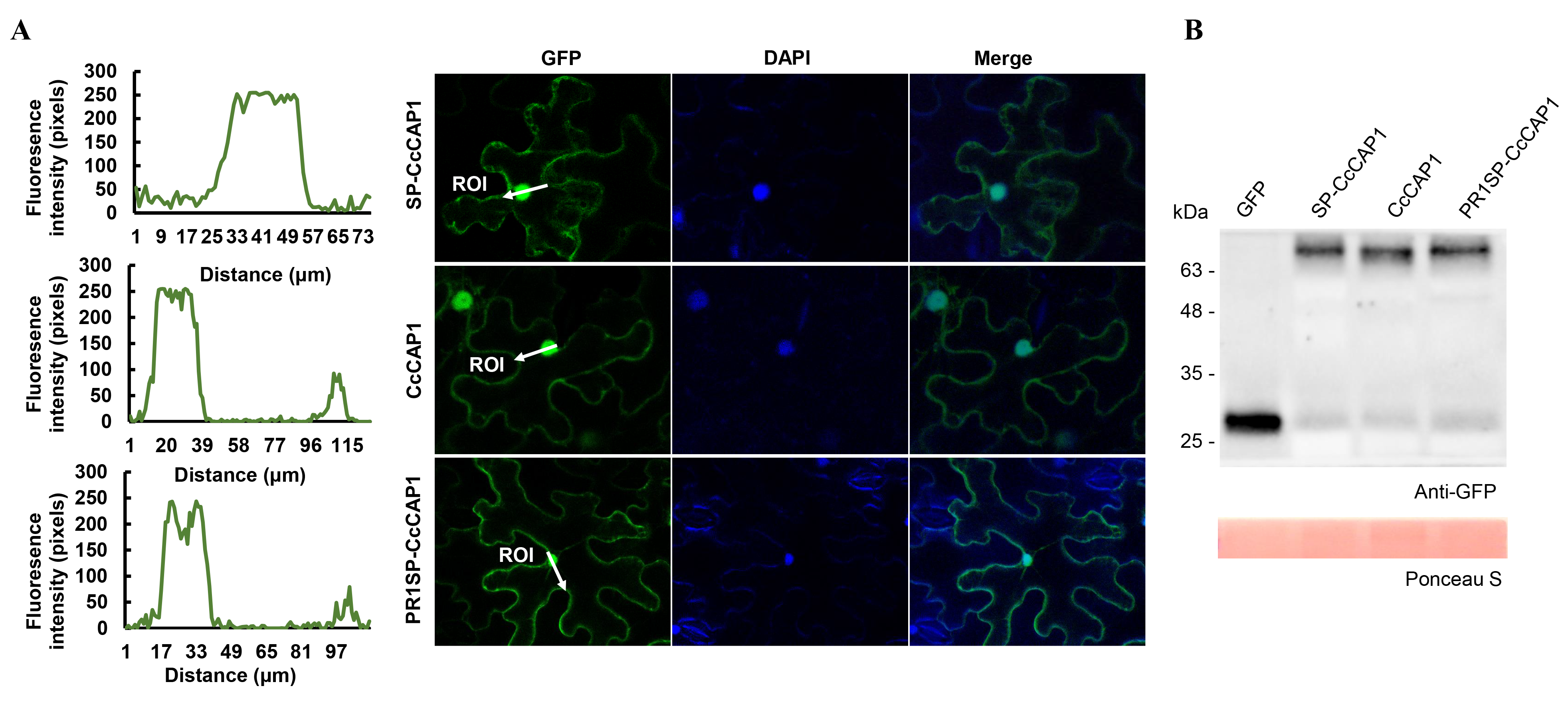

Supplement: FIG S4 [file msphere.00883-20-sf004.tif]
